# MicroRNAs Play a Role in Parkinson’s Disease by Regulating Microglia Function: From Pathogenetic Involvement to Therapeutic Potential

**DOI:** 10.3389/fnmol.2021.744942

**Published:** 2022-01-21

**Authors:** Silu Li, Guorong Bi, Shunchang Han, Rui Huang

**Affiliations:** ^1^Department of Rheumatology and Immunology, Shengjing Hospital of China Medical University, Shenyang, China; ^2^Department of Neurology, Shengjing Hospital of China Medical University, Shenyang, China

**Keywords:** microRNA, Parkinson’s disease, microglia, inflammation, therapy

## Abstract

Parkinson’s disease (PD) is a clinically common neurodegenerative disease of the central nervous system (CNS) characterized by loss of dopamine neurons in the substantia nigra. Microglia (MG), as an innate immune cell in the CNS, are involved in a variety of immunity and inflammatory responses in the CNS. A number of studies have shown that the overactivation of MG is one of the critical pathophysiological mechanisms underlying PD. MicroRNAs (miRNAs) are considered to be an important class of gene expression regulators and are involved in a variety of physiological and pathological mechanisms, including immunity and inflammation. In addition, miRNAs can affect the progress of PD by regulating the expression of various MG genes and the polarization state of the MG. Here, we summarize recent articles and describe the important role of MG pathological polarization in the progression of PD, the diverse mechanisms responsible for how miRNAs regulate MG, and the potential therapeutic prospects of miRNAs for PD. We also propose that the regulation of miRNAs may be a novel protective approach against the pathogenesis of PD.

## Introduction

Parkinson’s disease (PD) is one of the most common neurodegenerative diseases of the central nervous system (CNS) with an incidence of up to 1–2% among people over 60 years-of-age (Glass et al., 2010; Blaylock, 2017). PD has become one of the serious challenges facing today’s society (Ascherio and Schwarzschild, 2016). The significant pathological feature of PD is the loss of dopaminergic neurons in the substantia nigra pars compacta (SNpc) (Samii et al., 2004). In addition, the conversion of alpha-synuclein (α-syn) into oligomers and fibrils is the hallmark of a range of neurodegenerative disorders including PD (Galvagnion, 2017). However, the cellular and molecular mechanisms regulating the occurrence and development of PD remain unclear (Emamzadeh and Surguchov, 2018). There is increasing evidences that neuroinflammatory response is an important factor in the pathological process of PD (Kirkley et al., 2017). Postmortem analysis and PD models indicated that activated microglia (MG) and increased levels of proinflammatory factors were important pathological features of PD (Chen et al., 2008; Liu et al., 2020). MG, the resident macrophage in the CNS and play a vital role in the response of the brain injury (Sakuma et al., 2016). In fact, the activation of MG can remove excess neurotoxins, eliminate dying cells, and repair brain impairment (Lenz and Nelson, 2018). However, overactivated MG can be cytotoxic, producing and releasing excessive amounts of neurotoxic substances (including free radicals and pro-inflammatory cytokines) that eventually lead to neuronal impairment (Socodato et al., 2020). Studies have shown that MG are significantly active in the SNpc in PD mice, and that the secretion of inflammatory factors (TNF-α and IL-1β) is increased; these factors can attack the neurons and accelerate their degeneration and death, thus suggesting that activation of MG is one of the important links leading to the pathogenesis of PD (Bartels et al., 2020). Activated MG slowly releases pro-inflammatory cytokines, leading to the degeneration of dopaminergic neurons (Brudek, 2019). The continuous activation of the MG and the degeneration of neurons may form a vicious cycle that plays an important role in the degeneration of dopaminergic neurons (Joers et al., 2017). Therefore, accumulating studies have focused on the inhibition of MG activation to delay the progression of PD. For example, a number of studies in PD mouse models have shown that naloxone has neuroprotective effects by inhibiting MG activation and reducing the release of inflammatory factors (Liu et al., 2000, 2002; Lu et al., 2000). However, there are still many hurdles to overcome in applying these drugs to clinical practice.

MicroRNAs (miRNAs) are a class of evolutionarily conserved, endogenous, non-coding, and single stranded small RNAs. They bind to the 3′ UTR of target gene mRNA to mediate the degradation or translation inhibition of mRNA. miRNAs play a powerful role in the regulation of gene expression at the post-transcriptional level, mainly by inhibiting the translation or degradation of target protein (Li et al., 2013). miRNA is now recognized as a key participant in the process of cell development and plays a role in a series of physiological processes, including cell growth, proliferation, differentiation, aging, and apoptosis (Saliminejad et al., 2019). In addition, a large number of studies have demonstrated that changes in miRNA expression and function can lead to dysfunction of the immune system and regulate susceptibility to autoimmune diseases (Jin et al., 2018; Salvi et al., 2019). Studies have shown that miRNAs exhibit unique expression profiles in cells of the innate and adaptive immune system, and can regulate cell development and function (Mehta and Baltimore, 2016). In addition, abnormalities in the expression and function of miRNAs can lead to disorders in the immune system, thus leading to cancer and autoimmune diseases (Latini et al., 2017). To date, immune cells have been found to express more than 100 different miRNA molecules that can participate in molecular pathways that control the development and function of innate and adaptive immune responses (O’Connell et al., 2010). During the innate immune response, miRNAs such as miR-9 (Åkerblom et al., 2013), miR-21 (Kong et al., 2014), miR-29b (Thounaojam et al., 2014), and miR-34a (Alexandrov et al., 2013), exert important regulatory effects on MG. Moreover, as small molecules with biological activity, miRNAs can freely cross the blood-brain barrier (BBB). Therefore, it can be speculated that in the central-peripheral inflammatory network, miRNAs may form one of the material bases underlying the construction of the secondary network, thus creating a key pathway for communication between the central inflammatory state and the peripheral inflammatory state (Paschon et al., 2016).

In this review, we summarized the regulatory mechanisms that underlie the role of MG in the progression of PD and research relating to the potential of using miRNAs to regulate MG and thus treat PD. We believe that the regulation of miRNAs may represent a new way to prevent the pathogenesis of PD.

## The Microglial Response in Parkinson’s Disease

Microglia are an important component of glial cells, accounting for approximately 15% of glial cells (Lloyd and Miron, 2019). In a normal state, MG are in a resting state and mainly distributed in the CNS. The survival of MG depends on the colony-stimulating factor 1 receptor (CSF1R) signaling pathway and acts as an innate immune cell in the brain that can monitor changes in the surrounding microenvironment and pathogens (Song et al., 2018; Stephenson et al., 2018). MG exert phagocytic effects that can eliminate dead neurons and pathogens in the CNS (Chilakala et al., 2020). In addition, MG can also participate in neuronal remodeling by engulfing axons and myelin fragments (Cobourne-Duval et al., 2018). Under stress, the reactive MG bind to the Toll-like receptor (TLR) of a scavenger receptor complex to identify pathogens and remove necrotic tissue, pro-inflammatory factors, and neurotoxic factors (Janda et al., 2018). Studies have also shown that activated MG can exert a neuroprotective effect, although this depends on the degree of stimulation. Activated MG can be divided into a classic inflammatory type (M1 type) and an immunosuppressive type (M2 type) (Zhang et al., 2018). The M1 type can cause the release of destructive pro-inflammatory and chemotactic factors, aggravate the onset of inflammation, and induce brain impairment. The M2 type can be activated by interleukin-10 (IL-10) and bind to the fragment crystallized (Fc) receptor to form an immune complex that monitors apoptosis (Du et al., 2018; Lee and Suk, 2018).

Animal experiments and clinical studies have demonstrated significant levels of MG activation during the progression of PD, and that activated MG are morphologically enlarged and functionally stronger during the phagocytosis and secretion of pro-inflammatory factors (Phani et al., 2012). The up-regulated expression of major histocompatibility complex II (MHC II) and MG activation in PD were first confirmed in 1988 (McGeer et al., 1988). Further studies have shown that the elimination of MHC II in PD mice can reduce CNS lymphocyte infiltration, inhibit neuroinflammation, and exert a neuroprotective effect (Williams et al., 2018).

The pathological features of PD relate to the accumulation of α-syn, the formation of Lewy bodies, and the loss of dopaminergic neurons (Watanabe et al., 2020). Agglomerated α-syn can directly interact with MG through TLR2 and is internalized and concentrated in these cells (Kim et al., 2013). TLR2-mediated nuclear factor κB (NF-κB) promotes the release of inflammatory cytokines by regulating macrophage inflammatory protein (MIP) and monocyte chemotactic protein (MCP) (Ren et al., 2018). The NF-κB signaling pathway is involved in the activation of MG. NF-κB can promote the release of TNF-α, IL-1β, and type 2 interferon (IFN2), and the synthesis of inducible nitric oxide synthase (iNOS), thus enhancing the phagocytosis and migration of MG and leading to an increase of pathological α-syn and activation of MG. This accelerates the loss of dopaminergic neurons and aggravates the occurrence and development of PD (Huang C. et al., 2018; Huang Y. Y. et al., 2018). Inflammatory mediators can also increase the expression of fibroblast growth factor 20 (FGF20), mammalian target of rapamycin (mTOR), and early growth response factor 1(Egr-1), which induce neuroinflammation and accelerate the death of dopaminergic neurons by activating the TLR4-mediated transcription factor NF-κB signaling pathway (Yu et al., 2018). In addition, α-syn can promote activation of MG through MHC II, thus playing a role in the immune response and accelerating the progression of PD (Harms et al., 2013).

Microglia activation-induced neuroinflammation can also recruit peripheral monocytes to enter the CNS and differentiate into MG by knocking out the chemokine axis of C-C motif chemokine receptor 2 (CCR2)/C-C motif chemokine ligand 2 (CCl2) (Matsushima et al., 1994). In a pathological state, the effect of monocyte-derived macrophages on MG remains controversial. Some researchers believe that monocyte-derived macrophages can enhance inflammation and exacerbate disease progression (Ajami et al., 2011). Other researchers suggest that mononuclear macrophages play an anti-inflammatory role and promote tissue repair (Wattananit et al., 2016). The precise contribution of monocytes to PD also remains controversial. In the acute PD model, knockout of CCR2 can inhibit the entry of early monocytes into the CNS, while knockout of C-X3-C motif chemokine receptor 1 (CX3CR1) in MG or overexpression of CCl2 in astrocytes in MG induce more monocytes to enter the CNS and aggravate the impairment of dopaminergic neurons. Harms et al. (2018) suggested that in the α-syn-induced PD model, CCR2 knockout could inhibit the infiltration of mononuclear cells into the CNS and inhibit MG activation, thus playing a neuroprotective role.

With regards to the pathogenesis of inflammatory mediators, the overexpression of TNF-α can directly impair the CNS by inducing immune responses (Liddelow et al., 2017), and indirectly induce the synthesis of iNOS and cyclooxygenase 2 (COX-2); these latter factors can increase the production of NO and prostaglandin E2 (PGE2), thus enhancing the inflammatory response. TNF-α and IL-1β can cause BBB impairment, induce the synthesis of adhesion molecules and other inflammatory factors, and aggravate the inflammatory response of CNS. In addition, TNF-α and IL-1β can also induce the synthesis of one other, amplify the toxic effects of inflammatory factors, and promote the apoptosis of neurons (Zhao et al., 2007; Hernández-Romero et al., 2012).

Nucleotide-binding oligomerization domain-like receptor protein 3 (NLRP3) is an inflammatory complex that is present in MG (Wang et al., 2019). It leads to the continuous activation of MG in the presence of a persistent stimulus, and leads to the pro-inflammatory cytokines (IL-1β and IL18) production (Ramesh et al., 2013). Activated MG and NLRP3-rich inflammasomes have been shown to increase significantly in the substantia nigra of the midbrain in PD patients (Adams et al., 2019). In addition, polymerized α-syn can be captured by MG as an endogenous stimulus signal, thus activating NLRP3 inflammasomes and inducing cell pyroptosis (Alvarez-Erviti et al., 2011). The pathological development of PD includes the NLRP3 inflammasome-mediated secretion and maturation of IL-1β, and the activation of the NLRP3 inflammasome and its downstream molecule apoptosis-associated speck-like protein containing a CARD (ASC). Using a PD mouse model treated with the neurotoxin 1-methyl-4-phenyl-1, 2, 3, 6-tetrahydropyridin (MPTP), researchers found that the knockout of NLRP3 inhibited the progression of PD compared with wild-type mice (West et al., 2019). In the MPTP mouse model, the activation of the NLRP3 inflammasome in MG was shown to play a key role in the loss of dopaminergic neurons and the impairment of motor function; the application of an IL-1 receptor blocker significantly reduced the motor symptoms of the mouse model of PD (Lee et al., 2019). Moreover, the NLRP3 inflammasome has been shown to be significantly activated in the substantia nigra of PD patients; the components of the NLRP3 inflammasome in MG were also up-regulated, including NLRP3, ASC, and the effector molecule Caspase-1 (Haque et al., 2020). Therefore, activation of the NLRP3 inflammasome promotes the occurrence and development of PD. Interventional pathways involving the NLRP3 inflammasome and its related molecules may become a new treatment approach for PD.

**FIGURE 1:**
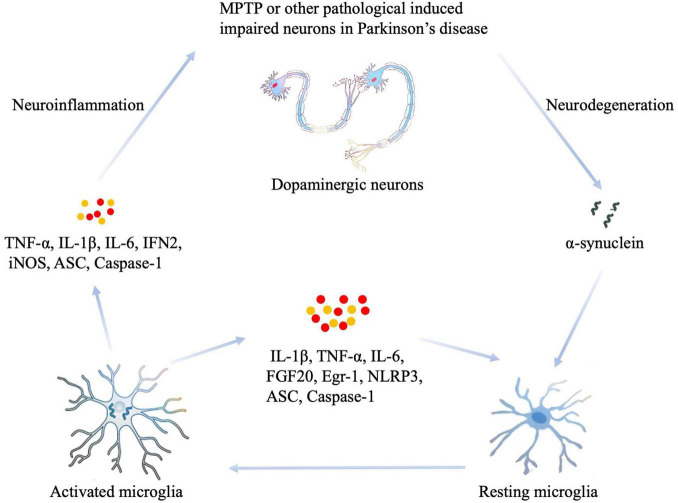
The role of MG in the neuroinflammation that occurs during PD. Damaged or dying neurons in PD, or toxin-induced models such as MPTP, cause the release of impair-associated molecular patterns (IAMPs) and α-synuclein to activate microglia through pattern recognition receptors (PRRs). Thus, the resting states of MG can be activated, thus leading to the eventual release of proinflammatory cytokines, including IL-1β, IL-6, TNF- α, and other inflammatory mediators such as ASC, IFN2, iNOS, and Caspase-1. In addition, increased levels of proinflammatory cytokines activate other resting MG, which induces neuroinflammation or neurodegeneration in the damaged area. MPTP, 1-methyl-4-phenyl-1,2,3,6-tetrahydropyridine; IL-1β, interleukin-1β; IL-6, interleukin-6; IFN2, type 2 interferon; iNOS, inducible NOS; TNF-α, tumor necrosis factor-α; ASC, apoptosis-associated speck-like protein containing a CARD.

In summary, damaged or dying neurons in PD, or toxin-induced models such as MPTP, cause the release of impair-associated molecular patterns (IAMPs) and α-syn to activate MG through pattern recognition receptors (PRRs). Thus, the resting states of MG can be activated, leading to the eventual release of proinflammatory cytokines, including IL-1β, IL-6, TNF- α, and other inflammatory mediators such as ASC, IFN2, iNOS, and Caspase-1. Moreover, increased levels of proinflammatory cytokines activate other resting MG, which induces neuroinflammation or neurodegeneration in the damaged area (Figure 1). Given the important association between neuroinflammation and MG in the pathological mechanisms underlying PD, targeting the neuroinflammation caused by MG activation has become a potential new target for the treatment of PD (Awogbindin et al., 2020). On the one hand, the reduction of neuroinflammation and neuronal impairment could be achieved by targeting the activation of MG as follows: (1) by targeting TLRs that trigger MG activation, such as TLR2, TLR4, and TLR9 (Li et al., 2012; Zhou P. et al., 2016; Maatouk et al., 2018); (2) by targeting key signaling pathways for MG activation, such as the NF-κB signaling pathway (Singh et al., 2020); and (3) by targeting pro-inflammatory factors such as TNF-α (Barcia et al., 2012). Furthermore, neuroinflammation can be inhibited by enhancing the M2 polarization state of MG; this could be achieved by (1) increasing the expression of anti-inflammatory factors in the CNS, for example, the injection of adeno-associated virus 2 to overexpress IL-10 in the brain of PD mice inhibited neuroinflammation and exerted a neuroprotective effect (Johnston et al., 2008; Joniec-Maciejak et al., 2014); and (2) inhibiting the polarization state transition from M1 to M2; for example, vitamin D promoted the transition of MG toward the M2 type, thus playing a neuroprotective role (Zhang X. et al., 2019). These studies on MG-targeted treatment have led to the improvement of PD symptoms, at least to some extent. However, a precision form of treatment has yet to be elucidated due to the heterogeneity of MG. Therefore, technologies and methods that target function-specific MG are still needed if we are to treat PD in a specific manner.

## MicroRNA

MicroRNAs (miRNAs) are a class of endogenous, non-coding, and small RNAs that are 20–25 base pairs in length and do not have an open reading frame (ORF) (Tafrihi and Hasheminasab, 2019). Mature miRNAs are derived from long precursors that fold into hairpin structures and are processed by Dicer enzyme or Dicer-like endonuclease. The main function of miRNA is to inhibit the expression of target mRNA or degrade mRNA by binding to specific sites of target mRNA so as to regulate the expression of endogenous genes at the post-transcriptional level (Deshpande et al., 2018). Thus far, two main regulatory mechanisms have been described: if mRNA and miRNA are highly complementary, the miRNA mediates mRNA cleavage, however, if mRNA is only partially complementary to miRNA, then the miRNA inhibits mRNA translation. Most plant miRNAs and target mRNAs in the ORF are highly matched and are predominantly mediated by cutting, thus leading to the degradation of the target mRNA. In animals, miRNA is not completely complementary to the target mRNA and mainly inhibits translation. Instead of reducing mRNA levels, the miRNA reduces the expression levels of corresponding proteins (Henshall et al., 2016).

Interestingly, miRNAs can also regulate the activation and polarization process of MG and affect the progression of a variety of neurological diseases. miR-155 is the most well-studied and defined miRNA with pro-inflammatory effects. In a rat model induced by red alginine, the overexpression of miR-155 was shown to enhance the activity of M1-type MG, inhibit the generation of BDNF, and aggravate brain impairment after epilepsy (Fu et al., 2019). However, in miR-155 knockout mice, the expression levels of MHC II and iNOS decreased significantly, while the M1 type polarization was also inhibited (Thome et al., 2016). miR-181 is a family of miRNAs that are highly expressed in the brain, including four types of miR-181 (a/b/c/d). Research in models of acute ischemic stroke has shown that miR-181a and miR-181c can up-regulate COX-1 levels, assist M1-type MG polarization, and promote both inflammatory responses and neuronal apoptosis (Ma et al., 2016). miR-301b can down-regulate the expression of the *NTPX2* gene, activate the NF-κB pathway, promote the polarization of M1 type MG, and increase the release of TNF-α (Saika et al., 2017). miR-125b can down-regulate the ubiquitin editing enzyme A20, activate the NF-κB pathway, induce the M1 polarization of MG in the amyotrophic lateral sclerosis model, and promote neuronal apoptosis (Parisi et al., 2016). Since M2-type MG can inhibit the inflammatory response and promote the repair of neuronal injury, the methods by which miRNA can induce M2-type polarization have become a major research focus over recent years. miR-124 was originally thought to be a brain-specific miRNA that regulates neuronal growth and differentiation, and can also regulate MG polarization and participate in the inflammatory response (Veremeyko et al., 2019). In a rat model of pilocarpine-induced epilepsy, miR-124 can inhibit cAMP response-element-binding protein (CREB), up-regulate the secretion of IL-10, and promote the conversion of M1 type MG to the M2 type (Wang et al., 2016). In spinal cord injury, miR-124 can also up-regulate Arg-1 and TGF-β, down-regulate the expression of iNOS and TNF-α through the C/EBP-α pathway, promote M2-type polarization, and exert a neuroprotective role (Yu et al., 2017). miR-145-5p can also promote M2-type polarization by inhibiting the release of TNF-α (Xie et al., 2017). Although the specific mechanism by which miRNAs promote M2 polarization is not yet fully understood, the anti-inflammatory and neuroprotective effects of M2-type MG indicate that miRNAs are a potential treatment for many neurological diseases.

## miRNAs and Microglial Responses in Parkinson’s Disease

Research has also focused on the ability of miRNAs to regulate the microglial response in PD, including their pathogenic roles and therapeutic values (Table 1). miR-124 has been reported to be a common brain-specific miRNA; Yao et al. (2019) showed that miR-124 was significantly down-regulated in an MPTP-induced mouse model of PD and could also inhibit neuroinflammation during the development of PD. In their study to explore the mechanisms underlying how miR-124 could inhibit neuroinflammation, the authors found that in the MPTP-induced PD mouse model, and in LPS-treated mouse MG line BV2 cells, the expression of sequestosome1 (p62) and phosphoric acid p38 mitogen-activated protein kinase (p-p38) were significantly increased. miR-124 therefore inhibits MG inflammation by targeting p62 and p38 in PD. The knockout of p62 in BV2 cells has been shown to prevent cell apoptosis and death in the human neuroblastoma cell line, SH-SY5Y, after MG activation. In addition, the exogenous delivery of miR-124 can inhibit the expression of p62 and p-p38 and attenuate the activation of MG in the SNpc of MPTP-treated mice, thus indicating that miR-124 can inhibit neuroinflammation in the development of PD by targeting p62, p38 (Yao et al., 2019). Moreover, the exogenous delivery of miR-124 can inhibit the expression of mitogen-activated protein kinase 3 (MEKK3) and p-p65 in the SNpc of mice treated with MPTP, and attenuate the activation of MG, thus suggesting that miR-124 may be a potential therapeutic target for regulating PD inflammatory response (Yao et al., 2018). miR-195 may also be one of the key factors that inhibits or stimulates inflammation during the development of PD. The expression of miR-195 was found to be decreased in LPS-stimulated BV2 cells, while the overexpression of miR-195 inhibited the release of proinflammatory cytokines, including iNOS, TNF-α, and IL-6, but induced the release of anti-inflammatory cytokines, including IL-4 and IL-10. Rho-associated kinase 1 (ROCK1) is known to be negatively regulated by miR-195; reduced ROCK1 expression also results in the same effect as the overexpression of miR-195 with regards to regulating the status of MG. miR-195/ROCK1 interactions are also known to exert a central role in inducing MG activation (Ren et al., 2019). Signal transducer and activator of transcription-3 (STAT3) was previously shown to be synchronously activated in the SNpc, while miR-let-7a targeted STAT3 and the expression of miR-let-7a was downregulated in a PD mouse. The overexpression of miR-let-7a has been shown to inhibit the activation of BV-2 MG and the production of pro-inflammatory factors induced by α-syn; effects that were eliminated by restoring STAT3 protein (Zhang J. et al., 2019). Therefore, miR-let-7a inhibits MG by targeting STAT3- mediated inflammation. Injecting miR-let-7a mimics into the mouse striatum was previously shown to inhibit the activation of MG and reduce the production of pro-inflammatory cytokines, thereby alleviating dyskinesia in PD mice that was induced by α-syn. As a negative regulator of neuroinflammation caused by MG, miR-let-7a may play a role in reducing the symptoms of PD (Zhang J. et al., 2019). miR-150 has been reported to be involved in the neuroinflammatory pathogenesis of PD. The AKT signaling pathway is very well known and plays a key role in many pathophysiological processes, including the progression of PD (Jha et al., 2015). Li et al. (2020) demonstrated that the expression of miR-150 was downregulated in PD patients and that the overexpression of miR-150 in LPS-treated BV2 cells could lead to the inhibited release of TNF-α, IL-1β, and IL-6. In addition, AKT3 was shown to be a direct target of miR-150 in BV2 cells; the overexpression of miR-150 may have a beneficial effect in PD by suppressing neuroinflammation by targeting AKT3 (Li et al., 2020). The expression of miR-330 was shown to be increased in an LPS-induced MG chronic neuroinflammatory model and an animal model of PD. The overexpression of miR-330 promoted the expression of SHIP1 and Arg1, inhibited the translocation of NF-κB and iNOS expression, and served to repress M1 polarization. miR-330 may negatively regulate the activity of NF-κB through *via* the MG SHIP1 target protein and continuously inhibit the polarization of MG induced by LPS *in vivo* and *in vitro*; this may be a promising neuroprotective strategy for the treatment of PD (Feng et al., 2021). SHIP1 is also a downstream target molecule of miR155-5p; this is one of the most important miRNAs and enables a robust inflammatory response. Reducing the expression of miR155-5p resulted in the upregulation of SHIP1 and the repression of NF-κB activity, thus leading to the inhibition of inflammation and MG activation (Feng et al., 2019).

**TABLE 1:** MiRNAs regulate microglial function involved in Parkinson’s disease.

miRNAs	miRNAs expression	Downstream targets	Regulatory effects on microglia	References
miR-124	Down-regulated	p62, p38, p-p65, MEKK3	Inhibit the expression of p62 and p-p38, and attenuate the activation of MG in SNpc	Yao et al., 2018, 2019
miR-195	Down-regulated	ROCK1	Inhibit the release of proinflammatory cytokines, including iNOS, TNF-α and IL-6	Ren et al., 2019
miR-let-7a	Down-regulated	STAT3	Inhibit MG by targeting STAT3 and reduce the production of pro-inflammatory cytokines	Zhang J. et al., 2019
miR-150	Down-regulated	AKT3	Inhibit the release of proinflammatory cytokines including TNF-α, IL-1β and IL-6	Li et al., 2020
miR-330	Up-regulated	SHIP1	Repress on M1 polarization, inhibit the translocation of NF-κB and iNOS expression	Feng et al., 2021
miR155-5p	Down-regulated	SHIP1	Repress NF-κB activity	Feng et al., 2019
miR-7116-5p	Down-regulated	TNF-α	Inhibit the production of TNF-α and the activation of MG	He et al., 2017
miR-7	Down-regulated	NLRP3	Inhibit the activation of NLRP3 inflammasomes	Zhou Y. et al., 2016
miR-190	Down-regulated	NLRP3	Inhibit the release of pro-inflammatory factors and activation of NLRP3	Sun et al., 2019
miR-29c	Down-regulated	NFAT5	Suppress pro-inflammatory cytokine release, NF-κB and NLRP3 inflammasome activation	Wang et al., 2020
miR-30e	Down-regulated	NLRP3	Inhibited α-synuclein protein expression and the activation of NLRP3 inflammasome	Li et al., 2018

Using a mouse model of PD induced by MPTP, He et al. (2017) demonstrated that miR-7116-5p plays a key role in MG-activated inflammation. miR-7116-5p was downregulated and found to target TNF-α. Furthermore, the enhancement of miR-7116-5p expression could inhibit the production of TNF-α and the activation of MG and prevent the loss of dopaminergic neurons (He et al., 2017).

It has been established that miR-7 expression decreases in PD patients; miR-7 targets α-syn in dopamine (DA) neurons and is associated with the pathophysiology of PD (Junn et al., 2009) Zhou Y. et al. (2016) found that NLRP3 is also a target gene of miR-7. Transfection of miR-7 was shown to inhibit the activation of NLRP3 inflammasomes in MG, while treatment with anti-miR-7 aggravated the activation of inflammasomes *in vitro*. The stereotactic injection of miR-7 mimics into the striatum of an MPTP-induced mouse model of PD attenuated the degeneration of dopaminergic neurons and improved the activation of MG. There is a direct relationship between miR-7 and NLRP3 inflammasome-mediated neuroinflammation in the pathogenesis of PD (Zhou Y. et al., 2016). Gaining a better understanding of the regulatory relationship between miR-7 and NLRP3 inflammasome may provide a new avenue for the treatment of PD. miR-190 has also been shown to be downregulated in LPS-induced BV2 cells; the overexpression of miR-190 could inhibit the release of pro-inflammatory factors, including TNF-α, TGF-β1, iNOS, and IL-6. There is a negative regulatory relationship between miR-190 and NLRP3. In an MPTP-induced mouse model of PD, miR-190 was shown to negatively regulate the expression and activation of NLRP3 to reduce neuronal damage and inhibit inflammation (Sun et al., 2019). miR-29c also exhibits anti-inflammatory properties in animal and neuronal models of PD. miR-29c was found to be decreased in LPS-induced BV-2 cells; the overexpression of miR-29c could suppress pro-inflammatory cytokine release, along with NF-κB and TXNIP/NLRP3 inflammasome activation. NFAT5 is a potential regulated target of miR-29c; miR-29c regulates NLRP3 inflammasomes by targeting NFAT5 and damages the inflammatory response of MG; consequently miR-29c may represent a promising target for the treatment of PD (Wang et al., 2020). miR-30e was previously shown to be significantly down-regulated in the SNpc of MPTP-induced PD mice (Li et al., 2018). The delivery of miR-30e agomir inhibited α-syn protein expression and attenuated the increase in inflammatory cytokines, including COX-2, TNF-α and iNOS. In addition, miR-30e also directly targeted NLRP3, thus suppressing the expression of NLRP3 mRNA and protein, thus inhibiting the activation of the NLRP3 inflammasome and the loss of dopamine neurons (Li et al., 2018).

## Prospects and Conclusion

Emerging evidence shows that the post-transcriptional regulation of miRNAs plays an important role in the regulation of gene expression (Majdi et al., 2016; Li et al., 2017). Several miRNAs have been suggested regulate α-SYN mRNA directly by binding its 3′UTR and negatively regulating its translation (Junn et al., 2009), *in vitro* SH-SY5Y PD cellular model that miR-34b and miR-34c were able to bind α-syn 3′UTR reducing its expression (Kabaria et al., 2015). *PRKN* and *PARK7* genes, encoding, respectively, for the proteins PARKIN and DJ-1, are both involved in the pathogenesis of autosomal recessive PD (Cookson, 2012). miR-34b and miR-34c were found to be downregulated in PD patients and may specifically regulate *PRKN* and *PARK7*, and significantly decreased the concentrations of PARKIN and DJ-1 proteins (Miñones-Moyano et al., 2011). Gain-of-function mutations in the *PARK8* gene can cause either familiar or sporadic PD, LRRK2 is an unusually large protein that in humans is encoded by the *PARK8* gene (Paisán-Ruíz et al., 2004). Upregulation of miR-184* and let-7 could attenuate the neurotoxic effects of mutant LRRK2, which suggested that LRRK2 may associate with PD pathogenesis *via* miRNA pathway modulation, therefore, highlighting new possible therapeutic strategies for PD (Gehrke et al., 2010).

As previously introduced, neuroinflammation is a major hallmark of PD. MG activation is clearly involved in the development of PD; miRNAs may affect the function of MG through multiple mechanisms, and have become new targets for the treatment of PD. However, there are still many problems that need to be addressed: (1) we need to identify methods to ensure that miRNAs can accurately regulate downstream mRNA to avoid off-target effects; (2) we need to understand miRNAs can be transported through the BBB without being degraded by lysosomes; and (3) each miRNA is often able to regulate multiple mRNAs; consequently, we need to be able to avoid adverse reactions if we are to treat PD by regulating MG response. Therefore, future studies should focus on the mechanisms used to deliver miRNAs so that miRNAs can play a greater role in the prevention and treatment of PD.

## Author Contributions

SL and GB acquired the information and wrote the preliminary draft. SH reviewed and revised the manuscript. RH revised details of the manuscript and provided the overall supervision. All authors contributed to the article and approved the submitted version.

## Conflict of Interest

The authors declare that the research was conducted in the absence of any commercial or financial relationships that could be construed as a potential conflict of interest.

## Publisher’s Note

All claims expressed in this article are solely those of the authors and do not necessarily represent those of their affiliated organizations, or those of the publisher, the editors and the reviewers. Any product that may be evaluated in this article, or claim that may be made by its manufacturer, is not guaranteed or endorsed by the publisher.
